# Workplace Perceptions and Experiences Related to COVID-19 Response Efforts Among Public Health Workers — Public Health Workforce Interests and Needs Survey, United States, September 2021–January 2022

**DOI:** 10.15585/mmwr.mm7129a3

**Published:** 2022-07-22

**Authors:** Rachel Hare Bork, Moriah Robins, Kay Schaffer, Jonathon P. Leider, Brian C. Castrucci

**Affiliations:** ^1^de Beaumont Foundation, Bethesda, Maryland; ^2^School of Public Health, University of Minnesota, Minneapolis, Minnesota.

The COVID-19 pandemic has strained many essential frontline professionals, including public health workers*; however, few studies have evaluated the specific challenges facing public health workers during this period. Data from the 2021 Public Health Workforce Interests and Needs Survey (PH WINS), a nationally representative survey of individual state and local governmental public health agency workers, provide insight into public health workers’ demographic characteristics and experiences during the COVID-19 pandemic, tenure, and intention to leave their organization^†^ (*1*). Surveyed governmental public health workers identified predominantly as non-Hispanic White (White), women, and aged >40 years; however, workforce characteristics differed by agency type. Overall, 72% of respondents reported working fully or partially in a COVID-19 response role at any point during March 2020–January 2022. An estimated 44% of workers reported that they were considering leaving their jobs within the next 5 years for retirement or other reasons. Of those considering leaving, 76% began thinking about leaving since the start of the COVID-19 pandemic. When asked what was needed, besides funding, to respond to the COVID-19 pandemic, 51% selected additional staff capacity. Survey findings highlight the importance of focused attention on recruitment and retention that promotes diversity (*2*) and workers with public health experience, which will be critical as the workforce rebuilds as the COVID-19 pandemic evolves.

All state health agencies, all large local health departments (LHDs) in the Big Cities Health Coalition,^§^ and a nationally representative sample of LHDs^¶^ with ≥25 staff members and serving a population of ≥25,000 were invited to participate in PH WINS. The survey was electronically administered using Qualtrics, a web-based survey tool, during September 13, 2021–January 14, 2022. A total of 137,446 surveys were distributed and completed by 44,732 persons (35% of eligible respondents); 9,106 surveys were excluded because they were returned as undeliverable or respondents were otherwise ineligible.** The final sample included 41,890 staff members^††^ from 47 state health agency central offices (SHA-COs), 190 large LHDs serving populations >250,000 (inclusive of Big Cities Health Coalition departments), and 249 medium LHDs serving populations of 25,000–250,000.

Data were cleaned, managed, and analyzed in STATA (version 17.0; StataCorp). Balanced repeated replication weights were constructed to account for the complex survey design and to adjust for nonresponse. Descriptive statistics were generated, and inferential comparisons were made using Rao-Scott design-adjusted chi-square testing (*3*). Analyses were conducted overall and stratified by agency type: SHA-CO, large LHD, and medium LHD. PH WINS 2021 was determined to be exempt from ongoing review by the NORC at the University of Chicago Institutional Review Board.

A majority of the governmental public health workforce self-identify as White (54%), women (79%), and aged >40 years (63%) (Table 1). Women make up 83% of the workforce at medium LHDs,^§§^ compared with 76% at SHA-COs, and 79% at large LHDs (p<0.001). The large LHD workforce had the most racial and ethnic diversity, with fewer than one half of workers (41%) identifying as White compared with two thirds at SHA-COs (66%) and medium LHDs (67%) (p<0.001). Employees who identify as White accounted for 66% of all executives in the state and local government public health workforce. Large LHD leadership is also more diverse, with 55% of large LHD executives identifying as White. In comparison, 78% of executives at medium LHDs and 74% of executives in SHA-COs identify as White (p<0.001).

**TABLE 1:** Characteristics of the governmental public health workforce — Public Health Workforce Interests and Needs Survey, United States, 2021

Characteristic	Weighted estimate, % (95% CI)			
	SHA-CO (n = 14,957)	Large LHDs* (n = 19,663)	Medium LHDs^†^ (n = 7,270)	National^§^ (N = 41,890)
Gender^¶,^**				
Women	75.7 (74.9–76.5)	78.5 (77.8–79.1)	82.9 (81.7–84.1)	**78.6 (78.1–79.0)**
Men	22.2 (21.5–23.0)	19.8 (19.2–20.5)	15.4 (14.3–16.7)	**19.7 (19.2–20.1)**
Identifies some other way	2.0 (1.8–2.3)	1.7 (1.5–1.9)	1.6 (1.3–2.0)	**1.8 (1.6–1.9)**
Race and ethnicity^¶,^**^,††^				
White	65.5 (64.6–66.3)	41.1 (40.3–41.9)	67.2 (65.6–68.7)	**53.7 (53.1–54.3)**
Hispanic or Latino	11.1 (10.5–11.7)	23.3 (22.6–24.0)	15.1 (13.9–16.5)	**18.0 (17.5–18.5)**
Black or African American	10.9 (10.4–11.5)	20.2 (19.5–20.9)	9.9 (8.9–10.9)	**15.3 (14.9–15.8)**
Asian	6.9 (6.5–7.4)	9.4 (8.9–10.0)	3.0 (2.4–3.7)	**7.4 (7.1–7.7)**
Two or more races	4.0 (3.7–4.4)	4.8 (4.4–5.1)	3.6 (3.0–4.2)	**4.3 (4.1,4.5)**
AI/AN	1.2 (1.0–1.4)	0.8 (0.7–1.0)	1.0 (0.7–1.4)	**0.9 (0.8–1.1)**
NH/OPI	0.4 (0.3–0.6)	0.4 (0.3–0.5)	0.2 (0.1–0.4)	**0.4 (0.3–0.5)**
**Age group, yrs^†^**				
≤21	0.1 (0.1–0.2)	0.2 (0.2–0.3)	0.3 (0.2–0.6)	**0.2 (0.2–0.3)**
21–30	11.0 (10.4–11.5)	13.7 (13.1–14.3)	14.7 (13.5, 16.0)	**13.1 (12.6–13.5)**
31–40	23.5 (22.7–24.3)	24.9 (24.1–25.7)	22.5 (21.2–23.8)	**24.0 (23.5–24.5)**
41–50	25.0 (24.2–25.8)	25.1 (24.3–25.9)	25.3 (23.9–26.8)	**25.1 (24.6–25.7)**
51–60	26.7 (25.9–27.5)	24.2 (23.5–25.0)	24.6 (23.3–26.0)	**25.0 (24.5–25.6)**
≥61	13.7 (13.0–14.4)	11.9 (11.4–12.5)	12.5 (11.5–13.6)	**12.6 (12.2–13.0)**
Tenure in current agency, yrs**				
0–5	49.0 (48.1–49.9)	50.2 (49.3–51.0)	49.9 (48.4–51.5)	**49.8 (49.2–50.4)**
6–10	18.8 (18.1–19.5)	16.3 (15.7–17.0)	15.2 (14.1–16.3)	**16.8 (16.4–17.3)**
11–15	11.7 (11.2–12.3)	11.2 (10.7–11.8)	11.8 (10.9–12.9)	**11.5 (11.1–11.9)**
16–20	8.9 (8.4–9.4)	9.2 (8.8–9.7)	9.6 (8.6–10.6)	**9.2 (8.9–9.6)**
≥21	11.6 (11.0–12.2)	13.0 (12.5–13.6)	13.5 (12.5–14.6)	**12.7 (12.3–13.1)**
Tenure in public health practice, yrs**				
0–5	33.4 (32.5–34.2)	36.4 (35.6–37.2)	39.5 (37.9–41.1)	**36.1 (35.5–36.7)**
6–10	19.9 (19.1–20.6)	18.5 (17.8–19.1)	17.2 (16.0–18.4)	**18.6 (18.2–19.1)**
11–15	14.1 (13.5–14.7)	13.7 (13.1–14.3)	13.2 (12.2–14.3)	**13.7 (13.3–14.1)**
16–20	11.6 (11.0–12.2)	11.4 (10.8–11.9)	11.1 (10.2–12.2)	**11.4 (11.0–11.8)**
≥21	21.1 (20.4–21.8)	20.1 (19.4–20.8)	19.0 (17.8–20.2)	**20.2 (19.7–20.6)**
Highest educational attainment**				
No college degree	10.9 (10.4–11.5)	15 (14.5–15.6)	19.9 (18.7–21.2)	**14.8 (14.4–15.2)**
Associates	9.2 (8.6–9.7)	10.5 (10.1–11.0)	17.2 (16.0–18.4)	**11.5 (11.1–11.9)**
Bachelor’s	35.3 (34.5–36.2)	37.3 (36.5–38.1)	39.8 (38.3–41.3)	**37.2 (36.7–37.8)**
Master’s	36.4 (35.5–37.2)	31.4 (30.6–32.2)	20.3 (19.0–21.6)	**30.6 (30.1–31.2)**
Doctorate	8.2 (7.7–8.7)	5.7 (5.3–6.1)	2.8 (2.3–3.4)	**5.9 (5.6–6.1)**
Public health degree (bachelor’s, master’s, or doctorate)**				
No	82.6 (82.0–83.3)	85.4 (84.8–86.0)	91.7 (90.7–92.5)	**85.9 (85.4–86.3)**
Yes	17.4 (16.7–18.0)	14.6 (14.0–15.2)	8.3 (7.5–9.3)	**14.1 (13.7–14.6)**
Supervisory status**				
Not a supervisor	70.3 (69.4–71.1)	73.2 (72.4–73.9)	76.3 (75.0–77.6)	**73.0 (72.4–73.5)**
Supervisor	17.5 (16.9–18.2)	16.7 (16.1–17.4)	14.9 (13.8–16.0)	**16.6 (16.2–17.0)**
Manager	10.2 (9.7–10.8)	8.0 (7.6–8.5)	5.5 (4.9–6.3)	**8.2 (7.9–8.5)**
Executive	2.0 (1.7–2.3)	2.1 (1.9–2.3)	3.2 (2.7–3.8)	**2.3 (2.1–2.5)**
Race and ethnicity among executives^¶,^**^,††^				
White	73.5 (67.2–79.0)	54.9 (49.3–60.3)	77.5 (68.1–84.7)	**66.3 (62.4–70.0)**
Hispanic or Latino	8.0 (5.0–12.6)	15.8 (12.1–20.5)	12.1 (6.5–21.4)	**12.7 (10.0–16.0)**
Black or African American	8.0 (5.3–12.0)	14.9 (11.5–19.2)	5.4 (2.4–11.9)	**10.4 (8.3–13.0)**
Asian	5.6 (3.4–9.1)	7.9 (5.5–11.2)	2.6 (1.1–5.7)	**5.8 (4.3–7.6)**
Two or more races	3.7 (1.6–8.7)	5.0 (2.8–8.5)	0.9 (0.3–3.1)	**3.5 (2.2–5.4)**
AI/AN	1.1 (0.3–3.5)	1.1 (0.4–3.0)	1.5 (0.2–9.9)	**1.2 (0.5–2.8)**
NH/OPI	0 (—)	0.4 (0.1–2.6)	0 (—)	**0.2 (0.0–1.2)**

**Abbreviations:** AI/AN = American Indian or Alaska Native; LHD = local health department; NH/OPI = Native Hawaiian or other Pacific Islander; SHA-CO = state health agency central office.

* Serving a population of >250,000.

^†^ Serving a population of 25,000–250,000.

^§^ Entire public health workforce.

^¶^ Sort order based on the largest to smallest percentage among the entire workforce (denoted as National).

** All estimates are significant at p<0.001.

^††^ White, Black, Asian, AI/AN, NH/OPI, and persons of two or more races were non-Hispanic; Hispanic or Latino persons could be of any race.

Respondents were relatively new to their agencies and to public health, with approximately 50% having been at their current agency for ≤5 years and 36% in public health practice for ≤5 years. Overall, 37% of respondents reported having a master’s or doctoral degree, with 14% reporting having a degree in public health.

Nationwide, 72% of respondents reported working in a COVID-19 response role during March 2020–January 2022 (Table 2). Approximately 80% of staff members at medium LHDs reported working on the COVID-19 response, compared with 75% at large LHDs and 62% at SHA-COs (p<0.001). Nationwide, 27% of the governmental public health workforce reported considering leaving their organization within the next 5 years for reasons other than retirement (Table 3). A total of 44% reported considering leaving their organization to retire or for other reasons; 76% began thinking about leaving since the start of the COVID-19 pandemic.^¶¶^ Apart from additional funding, the top three needs reported by workers to effectively respond to the COVID-19 pandemic in their jurisdiction were additional staff capacity (51%), more community support (30%), and more support from elected leaders (26%).***

**TABLE 2:** COVID-19 response role and needs among the governmental public health workforce — Public Health Workforce Interests and Needs Survey, United States, 2021

Characteristic	Weighted estimate, % (95% CI)			
	SHA-CO (n = 14,957)	Large LHDs* (n = 19,663)	Medium LHDs^†^ (n = 7, 270)	National^§^ (N = 41,890)
**COVID-19 response workforce role^¶^**				
Working on COVID-19 response	62.3 (61.4–63.1)	74.8 (74.0–75.5)	80.4 (79.0–81.7)	**72.1 (71.6–72.7)**
Reported needs^¶,^**				
Additional staff capacity (i.e., number or ability of staff members)	48.1 (47.1–49.0)	51.0 (50.1–51.9)	54.0 (52.4–55.6)	**50.7 (50.1–51.3)**
More community support	27.7 (26.8–28.5)	29.0 (28.3–29.8)	37.9 (36.4–39.4)	**30.4 (29.9–31.0)**
More support from elected leaders	28.5 (27.7–29.4)	24.1 (23.3–24.8)	25.2 (23.9–26.6)	**25.6 (25.1–26.1)**
Training	23.8 (23.0–24.7)	27.7 (26.9–28.5)	21.6 (20.3–23.0)	**25.3 (24.8–25.8)**
Better alignment with other sectors, such as businesses and schools	21.5 (20.8–22.3)	24.4 (23.7–25.2)	24.3 (23.0–25.6)	**23.5 (23.0–24.1)**
More support from agency leadership	20.1 (19.3–20.8)	21.7 (21.0–22.5)	16.5 (15.3–17.8)	**20.2 (19.7–20.7)**
Better messaging alignment with other leaders in my jurisdiction	17.5 (16.8–18.2)	18.9 (18.2–19.5)	16.2 (15.1–17.4)	**17.9 (17.5–18.4)**
Nonmonetary resources (i.e., know-how and equipment)	16.8 (16.1–17.6)	15.9 (15.3–16.6)	12.0 (11.0–13.2)	**15.4 (15.0–15.9)**
Other	11.6 (11.0–12.2)	9.7 (9.2–10.2)	8.2 (7.3–9.2)	**9.9 (9.6–10.3)**

**Abbreviations:** LHD = local health department; SHA-CO = state health agency central office.

* Serving a population of >250,000.

^†^ Serving a population of 25,000–250,000.

^§^ Entire public health workforce.

^¶^ All estimates are significant at p<0.001.

** In response to the question, “Besides funding, what do you need to respond to COVID?”

**TABLE 3:** Intent to leave the governmental public health workforce — Public Health Workforce Interests and Needs Survey, United States, 2021

Intent to leave organization	Weighted estimate, % (95% CI)			
	SHA-CO (n = 14,957)	Large LHDs* (n = 19,663)	Medium LHDs^†^ (n = 7,7270)	National^§^ (N = 41,890)
**Considering leaving within the next year (excluding retirement)^¶^**	28.3 (27.5–29.2)	28.2 (27.4–29.0)	21.5 (20.3–22.9)	**26.9 (26.4–27.4)**
**Considering leaving or retiring within the next 5 years^¶^**	46.0 (45.1–46.9)	44.9 (44.0–45.8)	39.8 (38.3–41.4)	**44.2 (43.6–44.8)**
**Length of time considering leaving^¶^**				
<3 mos	18.2 (17.0–19.4)	23.1 (21.9–24.4)	21.7 (19.5–24.2)	**21.3 (20.5–22.2)**
6–18 mos	27.5 (26.1–29.0)	26.2 (24.9–27.5)	29.7 (27.1–32.4)	**27.2 (26.3–28.1)**
Since before Mar 2020	26.8 (25.4–28.2)	22.5 (21.3–23.9)	21.7 (19.3–24.3)	**23.7 (22.8–24.6)**
**Considering leaving since start of COVID-19 pandemic (≤18 mos)^¶^**	73.2 (71.8–74.6)	77.5 (76.1–78.7)	78.3 (75.7–80.7)	**76.3 (75.4–77.2)**

**Abbreviations:** LHD = local health department; SHA-CO = state health agency central office.

* Serving a population of >250,000.

^†^ Serving a population of 25,000–250,000.

^§^ Entire public health workforce.

^¶^ All estimates are significant at p<0.001.

## Discussion

The findings from the 2021 PH WINS survey show that the public health workforce is not as racially and ethnically diverse as the constituents its services target, the workforce perceived more staff members with public health experience were needed to effectively respond to the COVID-19 pandemic, and many public health workers reported an intention to leave their organization in the near future. This finding is concerning, given a recent report that found approximately 80,000 additional full-time staff members are needed throughout the nation’s public health agencies to provide foundational public health services (*4*). The public sector faces similar challenges to the public health workforce, with reported increases in voluntary turnover, the need for more staff members to reduce workloads, and increased workplace stress (*5*). With the public health system facing immense pressure because of the prolonged COVID-19 response, worsening national health, increased stress, and burnout (*6*), potential significant staff losses would further strain an overtasked workforce.

 The governmental public health workforce is more racially and ethnically diverse than is the overall U.S. workforce (i.e., 77% White)^†††^; however, White employees are still the majority in all state and local health departments (54%). Further, this group remains overrepresented among public health executives, except for large LHDs, which have the most diversity in executive leadership (55% White executives). Diversity at all supervisory levels can facilitate a fuller understanding of the needs of culturally diverse communities (*7*). The disproportionate impact that COVID-19 has had on racial and ethnic minority communities (*8*) underscores the importance of a highly diverse workforce that can better fulfill the essential and emergent needs of all communities (*9*).

Nearly three quarters of respondents reported being deployed for the COVID-19 response. It is unclear what impact the necessary diversion of these resources had on other public health focus areas, many of which, including smoking, alcohol use, and violence, were likely exacerbated during the pandemic (*8*).

With nearly one half of the survey respondents having worked for ≤5 years at their current agency, and approximately one third having been in practice for ≤5 years, findings indicate that the COVID-19 pandemic might have been many employees’ first experience with a public health emergency. This finding coupled with the high percentage of the workforce reporting an intention to leave their organization might result in agencies with limited institutional knowledge from the COVID-19 pandemic response for future emergencies.

To grow and diversify the workforce in the face of potentially substantial turnover, agencies should consider redoubling efforts to increase and formalize recruitment pathways between academia and public health. Although hiring surges provide extra capacity, the workforce does not necessarily have the knowledge and expertise needed for an effective pandemic response. Recruitment and retention efforts should emphasize the need to retain knowledgeable and skilled employees with public health experience. Agencies might also want to address stress, burnout, and workplace environment factors^§§§^ (*10*).

The findings in this report are subject to at least four limitations. First, agency nonparticipation and individual nonresponse might pose limitations to generalizability; however, balanced repeated replication weights were applied to account for nonresponse and complex sampling. Second, the survey responses are largely self-reported, with inherent potential for biases, including social desirability bias. To mitigate potential bias, the study used previously used items where possible, and employed cognitive interviews and pretests for new items. Third, the study did not assess specific reasons for seeking leadership roles or retirement from the public health workforce by sociodemographic characteristics. Finally, the survey is of staff members who remained in the workplace, not those who had left. Although the prevalence of an intent to leave is comparable with that identified in previous administrations of PH WINS, actual turnover is plausibly much higher.

PH WINS provides a snapshot of the public health workforce during a period of unprecedented and prolonged emergency response. It is critical that workforce development efforts prioritize purposeful succession planning and recruitment and retention efforts that increase diversity as the workforce fortifies and rebuilds after the COVID-19 pandemic.

**Summary**

**What is already known about this topic?**

The COVID-19 pandemic has strained many essential frontline professionals, including public health workers.

**What is added by this report?**

The 2021 Public Health Workforce Interests and Needs Survey recorded the perspectives of the governmental public health workforce. During March 2020–January 2022, 72% of the workforce fully or partially served in a COVID-19 response role. Apart from funding, 51% of respondents cited a need for additional staff capacity to respond to COVID-19. Approximately 40% of the workforce intends to leave their jobs within the next 5 years.

**What are the implications for public health practice?**

Purposeful succession planning and focused attention on recruitment and retention that promotes diversity will be critical as the workforce rebuilds while the COVID-19 pandemic evolves.

## Footnotes

*
https://www.hhs.gov/surgeongeneral/priorities/health-worker-burnout/index.html

† PH WINS was first fielded in 2014 and a second time in 2017. PH WINS 2021 has five major domains: 1) workplace environment, 2) COVID-19 response, 3) training needs, 4) addressing public health issues, and 5) demographics.

§
https://www.bigcitieshealth.org

¶ LHDs for the nationally representative sample were contributed in one of four ways: 1) Probability: a stratified, clustered random sample based on a list of all eligible LHDs. Strata were constructed based on cross-classification of 10 U.S. Department of Health and Human Services (HHS) regions and two population sizes (25,000–250,000 and >250,000); 2) Certainty: LHDs that participated in 2017 through the random sample were invited to participate in the 2021 survey; 3) Certainty: local staff members who participated as an employee of an LHD in a nondecentralized state. Decentralization is a governance structure that refers to the relationship a state health agency has with the LHDs in their state. The four types of governance are decentralized, centralized, mixed, and shared; and 4) Certainty: LHDs that participated through the PH WINS for All pilot program, a partnership with the Region 5 Public Health Training Center and Northwest Center for Public Health Practice’s Region 10 Public Health Training Center aimed at recruiting all local health departments in each region (Region 5: Illinois, Indiana, Michigan, Minnesota, Ohio, and Wisconsin; Region 10: Alaska, Idaho, Oregon, and Washington, including those with <25 staff members and serving a population of <25,000. These small LHDs participating through PH WINS for All were excluded from the LHD sample.

** Persons whose emails were returned as undeliverable or failed or those who left their organizations were considered ineligible.

†† A total of 2,842 staff members were excluded from the nationally representative sample: 1,803 staff members work in LHDs that have <25 staff members and serve <25,000 persons; 1,039 staff members were excluded for other reasons.

§§ Large LHDs serve a population >250,000. Medium LHDs serve a population of 25,000–250,000.

¶¶ Survey completion ranged from September 2021 to January 2022. Eighteen months earlier coincides with the start of the COVID-19 pandemic.

*** Response options to the question, “Besides funding, what do you need to respond to COVID?” were the following: more support from agency leadership; nonmonetary resources (i.e., know-how and equipment); additional staff capacity (i.e., number or ability of staff members); training; more community support; more support from elected leaders; better messaging alignment with other leaders in my jurisdiction; better alignment with other sectors, such as businesses and schools; and other (please specify).

†††
https://www.bls.gov/opub/reports/womens-databook/2021/home.htm

§§§
https://debeaumont.org/wp-content/uploads/dlm_uploads/2022/03/Stress-and-Burnout-Brief_final.pdf
